# Three-Dimensional Visualization of Myocardial Ischemia Based on the Standard Twelve-Lead Electrocardiogram

**DOI:** 10.1155/2016/7697980

**Published:** 2016-06-28

**Authors:** Ying Ma, Yang Sheng, Tian Ruixia, Chen Xun

**Affiliations:** ^1^Department of Precision Machinery and Precision Instrumentation, University of Science and Technology of China, Hefei, Anhui 230027, China; ^2^The 105th Hospital of PLA, Hefei, Anhui 230031, China

## Abstract

A novel method was proposed for transforming the ischemic information in the 12-lead electrocardiogram (ECG) into the pseudo-color pattern displayed on a 3D heart model based on the projection of a ST injury vector in this study. The projection of the ST injury vector at a point on the heart surface was used for identifying the presence of myocardial ischemia by the difference between the projection value and the detection threshold. Supposing that myocardial ischemia was uniform and continuous, the location and range of myocardial ischemia could be accurately calculated and visually displayed in a color-encoding way. The diagnoses of the same patient were highly consistent (kappa coefficient *k* = 0.9030) between the proposed method used by ordinary people lacking medical knowledge and the standard 12-lead ECG used by experienced cardiologists. In addition, the diagnostic accuracy of the proposed method was further confirmed by the coronary angiography. The results of this study provide a new way to promote the development of the 3D visualization of the standard 12-lead ECG, which has a great help for inexperienced doctors or ordinary family members in their diagnosis of patients with myocardial ischemia.

## 1. Introduction

It is an important prerequisite for patients with myocardial ischemia to get timely and effective treatment that their information of myocardial ischemia can be obtained accurately and reliably [1–4]. The 12-lead ECG is widely used in ambulances, emergency rooms, outpatient examination rooms, hospital wards, and health centers as a convenient, rapid, effective, and low-cost means of cardiac inspection [5–7]. However, the diagnostic criteria widely adopted for the diagnosis of myocardial ischemia according to the 12-lead ECG are based on the ST segment deviation and very complex, so some medical professionals lacking experience find it hard to master it very well, not to mention ordinary people without medical knowledge [8, 9]. Unfortunately, misdiagnoses and missed diagnoses often occur in clinical practice. The visualization of the information of myocardial ischemia in the 12-lead ECG as well as the simplification of its diagnostic criteria is the dream of many researchers in this field. The advantage of this kind of improvement is that it can reduce the rate of misdiagnosis and missed diagnoses of medical professionals; in addition, it can also gradually make the 12-lead ECG into homes, so that ordinary people can diagnose myocardial ischemia for themselves.

The visualization methods of the 12-lead ECG can be divided into two kinds, two-dimensional methods and three-dimensional methods. The ST compass is a method of two-dimensional display [10, 11], which consists of two circular patterns, vertical and horizontal, respectively. The location and range of myocardial ischemia are marked out with color in the corresponding sectors on the two circular patterns. However, it is not a simple thing for the same heart to link its two circular patterns of the ST compass to its three-dimensional shape. The diagnosis of myocardial ischemia with the ST compass requires specialized training of medical professionals, which is not easy for ordinary people to grasp. A method was proposed by Zizzo and his colleagues for calculating the three-dimensional potential distribution on the epicardium including the information of myocardial ischemia by using the finite element method [12, 13]. The surface material of epicardium was assumed to be isotropy, and 2500 square elements were used to mesh the front and left-hand side of the heart's surface, in which the square elements corresponding to the 12-lead ECG were regarded as an ideal voltage source. This method made the presentation of the information relating to acute ischemia intuitive and meaningful, and it was particularly helpful to clinicians in drawing attention to onset conditions that are clinically relevant to initiate prompt treatment. However, the mathematical model for finite element calculation was based on a finite set of diagnostic data of a few patients suffering from acute ischemia, so it needs more experimental data to prove its versatility in diagnosis and display of myocardial ischemia. On the other hand, the calculation of three-dimensional potential distribution on the epicardium was only implemented on the partial surface of the heart related to its left ventricle, which made the display of acute ischemia on heart model incomplete.

In order to realize the visualization of the 12-lead ECG, we developed a novel method for displaying information relating to myocardial ischemia on a 3D heart model created by scanning a physical model of the heart. The central point of the region under ischemia is accurately located by the orientation of ST injury vector. Meanwhile, the equivalent region of myocardial ischemia is obtained by the magnitude of the ST injury vector under the hypothesis of uniformity and continuity of ischemic distribution in the circular region. The proposed method is generally applicable, as it does not theoretically and technically need any special restriction on patients to be detected. The experimental results of the proposed method will be compared to the diagnostic results of experienced cardiologists and the detection results of coronary arteriography to check the feasibility and validity of the method in this paper. Using this new method, experienced professionals can more quickly and accurately diagnose the degree and range of myocardial ischemia; inexperienced novices can decrease their misdiagnosis and missed diagnosis; ordinary people can perform self-diagnosis so as to see a doctor in time.

## 2. Materials and Methods

### 2.1. Digital 3D Modeling of a Heart

**Figure 1:**
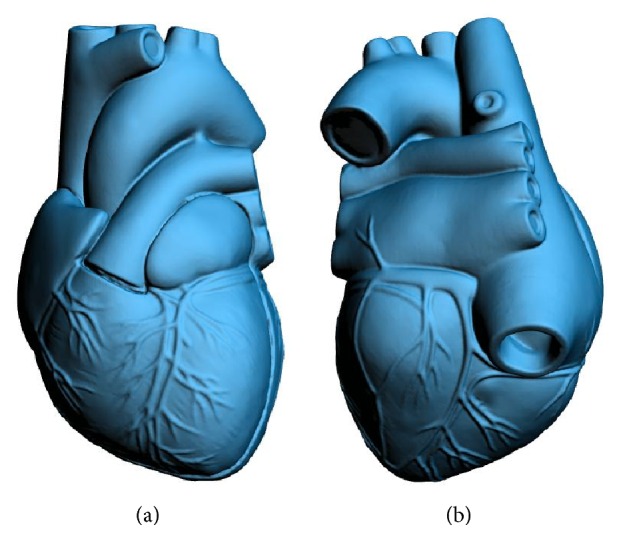
Front and back views of the digital 3D heart model.

A good 3D heart model is essential for the correct 3D display of the information of myocardial ischemia in the 12-lead ECG [14–16]. To achieve that, we used a standard medical heart model and got the digital 3D heart model by reverse engineering. First, the standard medical heart model was scanned by using a high-resolution 3D scanner (OptimScan-5M, SHINING 3D, China). The specific modeling process is divided into three steps, namely, calibration, scanning, and postprocessing. In order to ensure the accuracy of the 3D scanner we must calibrate it with a standard three-dimensional sphere. The standard medical heart model needs to be coated with white reflective powder and then scanned, because some of the light absorbing surface material is not conducive to the acquisition of 3D scanning data and ultimately affects the accuracy of three-dimensional reconstruction. In addition, artificial marking points should also be used to help the 3D scanner correct the distortion of the reconstruction model. The upper semimodel and the complex shape of blood vessels were obtained by means of extrusion, and the lower semimodel including the apical part was obtained by curve rotation. The 3D reconstruction result of the scanner needed to be smoothed by the software Geomagic Studio, and finally the high quality digital 3D heart model was obtained as shown in Figure 1. The model surface is smooth, its structure and the distribution of the coronary artery are clear at a glance. The origin of the three-dimensional coordinate system was built at the center-point of the left ventricle; then the region of myocardial ischemia could be marked on the 3D heart model with UGNX software. The full data of the model are stored in a STL format file, in which the outline of the heart model consists of a set of triangular elements. Thus, the digital 3D heart model we created is the true representation of the human heart. Through software programming, one can easily change its viewing angle, partition a specific region on its surface, and change the color of its surface.

Accurate positioning of the center point of the left ventricle is a prerequisite for establishing a three-dimensional coordinate system. The trouble is that the shape of the left ventricle is a complex conical cavity. Therefore, the position cannot be simply identified as a regular geometric body. We need to know the three-dimensional geometry of a left ventricular cavity to further get the coordinates of the center point of the left ventricle. The left ventricular cavity is converted into a solid model with filling materials due to the difficulty of scanning cavity with a 3D scanner and the poor quality of its 3D reconstruction. The center position of the solid model is considered to be that of the left ventricle. The center point coordinates of the entity model are regarded as those of the left ventricle. The center coordinates of a solid model can be calculated as

(1) xc=∮Sx ds∮Sds,yc=∮Sy ds∮Sds,zc=∮Sz ds∮Sds,

where *x*_*c*_, *y*_*c*_, and *z*_*c*_ are the three-dimensional coordinates of the center point, respectively. The surface finite elements can be replaced as the micro triangles, as the surface obtained by scanning is composed of a large number of micro triangles. As a result, (1) can be derived as

(2) xc=∑x·SΔ∑SΔ,yc=∑y·SΔ∑SΔ,zc=∑z·SΔ∑SΔ,

where *S*_Δ_ is the area of a micro triangle. The final coordinates of the center point depend on the coordinate system and the software used for processing.

After determining the origin of the coordinate system, we must continue to determine the direction of *XYZ* three coordinate axes. An object has six degrees of freedom in the three-dimensional space, which can be constrained by three non-identical-plane points. The lead I, −aVF, and *V*_2_ were used as the direction of the axes *x*, *y*, and *z*, respectively. It should be noted that although five experienced cardiologists participated in the establishment of the coordinate system, it is still difficult to ensure that the directions of axes are absolutely correct. The origin and coordinate system need to be further calibrated, which will be described in the following.

### 2.2. Synthesis of the ST Injury Vector

The ST injury vector is obtained from the 12-lead ECG. We designed a portable 12-lead ECG acquisition and processing system, which consists of leads, a pretreatment circuit, an ECG amplification-acquisition unit, a communication interface, and so forth. The pretreatment circuit mainly protects its following circuit and reduces the external electromagnetic interference. The ADS1298 (TI, Texas, America) and the proASIC3 (ACTEL company, New York, America) were used as the core chips of the amplification-acquisition unit. A conversion module for RS-232 to USB was used as a communication interface, which was responsible for transmitting the ECG data to a personal computer.

The ST injury vector was calculated by the ST deviation of each lead of the 12-lead ECG. Therefore, it is very important to accurately get the ST deviation. The ST segment measurement point is at the 108 ms after the R wave crest and the isopotential reference point at the 80 ms before the R wave crest. The potential difference between two points is defined as the ST segment deviation Δst [17–19].

**Figure 2:**
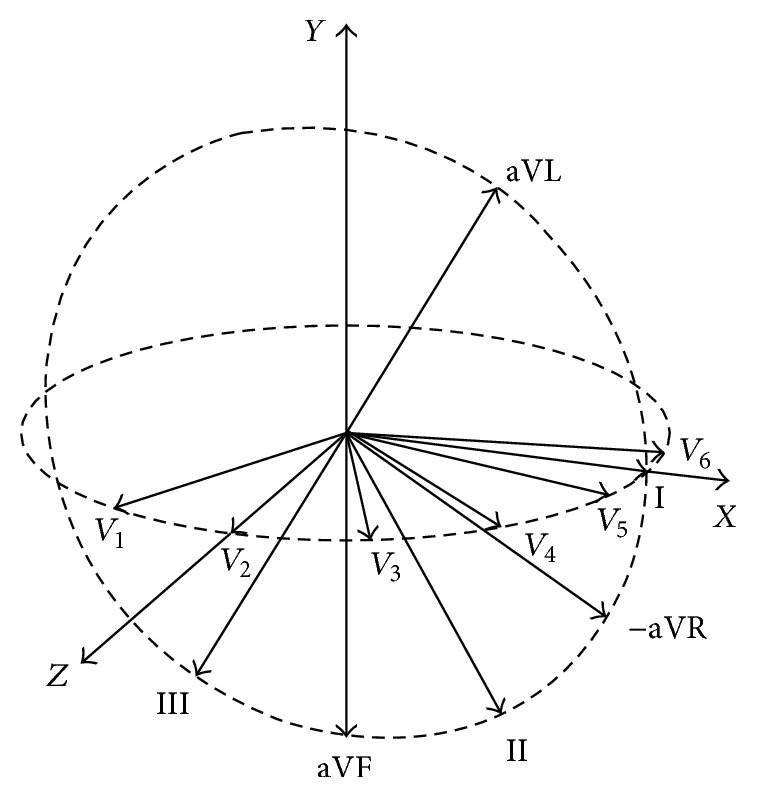
Spatial distribution of the lead subvectors on the frontal and horizontal plane.

A ST injury vector could be decomposed into two orthogonal subvectors, ${\vec{v}}_{h}$ on the horizontal plane and ${\vec{v}}_{p}$ on the frontal plane. The lead subvectors used for the synthesis of ${\vec{v}}_{h}$ and ${\vec{v}}_{p}$ were distributed on the above two planes, respectively. The vectors (${\vec{\alpha }}_{1},{\vec{\alpha }}_{2},\ldots ,{\vec{\alpha }}_{6}$) corresponding to limb leads and augmented limb leads (I, II, III, aVR, aVL, aVF) were distributed on the frontal plane, while the vectors (${\vec{\alpha }}_{7},{\vec{\alpha }}_{8},\ldots ,{\vec{\alpha }}_{12}$) corresponding to chest leads (*V*_1_, *V*_2_,…, *V*_6_) distributed on the horizontal plane [20–23]. In the orthogonal *XYZ* coordinate system, the *x*-*y* plane is the frontal plane and the *x*-*z* plane is the horizontal plane. It is worth noting that all vectors and subvectors are starting from the center point of the left ventricle. Respectively (see Figure 2), the limb-lead vectors (${\vec{\alpha }}_{1},{\vec{\alpha }}_{2},\ldots ,{\vec{\alpha }}_{6}$) and augmented-limb-lead vectors point to 0° (I), 60° (II), 120° (III), 30° (−aVR), −30° (aVL), and 90° (aVF) on the *x*-*y* plane; and the chest-lead vectors (${\vec{\alpha }}_{7},{\vec{\alpha }}_{8},\ldots ,{\vec{\alpha }}_{12}$) point to 115° (*V*_1_), 90° (*V*_2_), 65° (*V*_3_), 40° (*V*_4_), 15° (*V*_5_), and −10° (*V*_6_) on the *x*-*z* plane.

As mentioned previously, the origin of the *XYZ* coordinate system was located at the center point of the left ventricle; however, our follow-up study found that it was in conflict with the axis positions identified by the panel of experts. The three coordinate axes of the *XYZ* coordinate system were not perpendicular to each other. Therefore, the origin needed to be adjusted to calibrate the *XYZ* coordinate system to ensure that its three-axes are in the right direction. It should be noted that such a calibration of the *XYZ* coordinate system is only in line with the definition of the *XYZ* orthogonal coordinate system, and it is easy to get the recognition of most medical experts but may not be consistent with the laws of cardiac electrophysiology. For the ST injury vector, this kind of artificial inconsistency is equivalent to the transformations of translation and rotation. In addition, the human heart and body are not spherical, so those electrodes are different in the distance to the heart, which will change the size and direction of the ST injury vector. This means that the theoretical calculation of the ST injury vector also needs to be calibrated with a PET or other “gold standard” equipment. We choose the PET image and use it to calibrate the ST injury vector. In this paper, the weighting coefficient *e*_*i*_ (*i* = 1,2,…, 12 denotes leads I, II, III, aVR, aVL, aVF, *V*_1_, *V*_2_, *V*_3_, *V*_4_, *V*_5_, *V*_6_, resp.) for the ST segment deviation Δst_*i*_ is expressed as

(3) $$\begin{array}{c} e_{i}=\left\{\begin{array}{cc} 1 & \Delta st_{i}>0,\;i\neq 7,8,9,\\ \frac{1}{3} & \Delta st_{i}>0,\;\;i=7,8,\\ \frac{1}{5} & \Delta st_{i}>0,\;\;i=9,\\ 2 & \Delta st_{i}<0. \end{array}\right. \end{array}$$

The projection length of the ST injury vector $\vec{v}$ in the direction of vector ${\vec{\alpha }}_{i}$ can be calculated as

(4) $$\begin{array}{c} l_{i}=\left|\;\Delta st_{i}\;\right|\cdot e_{i}=\vec{v}\cdot {\vec{\alpha }}_{i}, \end{array}$$

where *l*_*i*_ (*i* = 1, 2,…, 6) is the projection length of ${\vec{v}}_{p}$ in the direction of vector ${\vec{\alpha }}_{i}$ (*i* = 1, 2,…, 6) while *l*_*i*_ (*i* = 7, 8,…, 12) is the projection length of ${\vec{v}}_{h}$ in the direction of vector ${\vec{\alpha }}_{i}$ (*i* = 7, 8,…, 12). The coordinates of a unit vector calculated as ${\vec{\alpha }}_{i}/l_{i}$ (*i* = 1, 2,…, 6) in *x*-*y* plane are (*x*_*i*_, *y*_*i*_) while those of ${\vec{v}}_{p}$ are (*x*_*p*_, *y*_*p*_, 0). The least square method was used for minimizing the error induced by the static indeterminateness of the inverse projection equations

(5) x1⋮x6y1⋮y6Tx1⋮x6y1⋮y6xpyp=x1⋮x6y1⋮y6Tl1⋮l6.

The result of (5) is the vector ${\vec{v}}_{p}$, and the calculation of ${\vec{v}}_{h}$ is identical with it. The ST injury vector $\vec{v}$ is obtained by

(6) $$\begin{array}{c} \vec{v}={\vec{v}}_{p}+{\vec{v}}_{h}. \end{array}$$

Through the above steps, the best ST injury vector is completely determined.

### 2.3. Display of Myocardial Ischemia

In our method, the myocardial ischemic region cannot be truly reproduced as a PET image due to the limited information provided by the 12-lead ECG. The central point of the myocardial ischemic region is relatively easy to be determined, that is, the intersection point of the ST injury vector with the heart surface. The regional distribution of myocardial ischemia is complicated. We have to simplify the calculation of the regional distribution because there is only one parameter, the magnitude of the ST injury vector, available. We use the ST injury vector as the axis and *r* as the radius to make a circular cylinder; then the intersection line of the cylindrical outer circle and the surface of the heart is the boundary line of the regional myocardial ischemia. It is needed to point out that the myocardial ischemia is assumed to be uniformly distributed within this equivalent region. The radius *r* is specifically calibrated by PET images. We chose the scheme for pseudo-color display as follows: normal myocardium was in red and the ischemic myocardium in blue purple.

### 2.4. Protocol for the Clinical Validation

The purpose of clinical validation is to explore the feasibility of the diagnosis of myocardial ischemia by ordinary people without medical knowledge based on the method of 3D heart model display. The specific contents validated were the comparisons with the experienced cardiologists' diagnoses according to the standard 12-lead ECG and coronary angiography images. Both patients with coronary heart disease and healthy people were selected as subjects and mixed into a few groups. The control clinical trials were designed with double blind method and the results were statistically analyzed by Kappa. The equipment used for the clinical trials was a FX-7500 ECG machine (Fukuda, Tokyo, Japan) and a digital subtraction angiography (GE, America). The clinical trials were carried out at the 105th hospital of the People's Liberation Army. The study protocol was approved by the institutional review board of the University of Science and Technology of China, Hefei, China, and all patients provided written informed consent.

## 3. Results and Discussion

**Figure 3:**
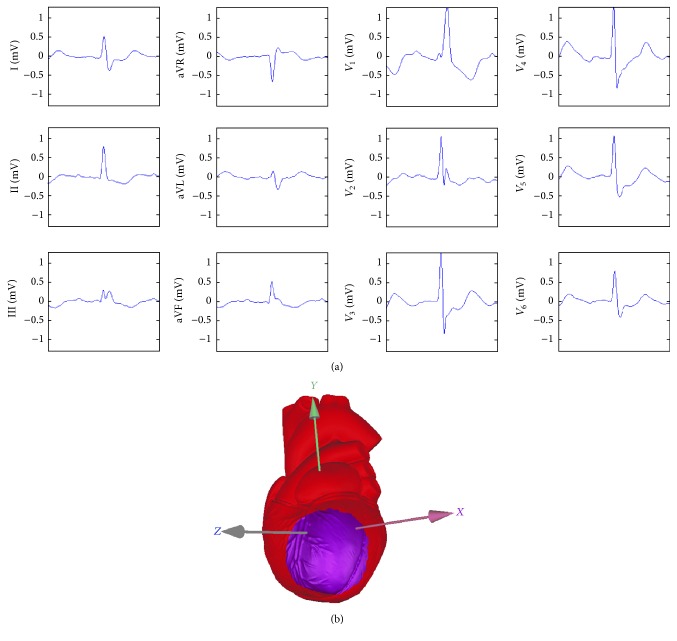
Results of a 69-year-old female patient. (a) Her 12-lead ECG. (b) Her pattern of myocardial ischemia displayed on a 3D heart model.

48 patients randomly selected from the total subjects, 25 males and 23 females, age from 46 to 93 years, were diagnosed by ordinary people without medical knowledge and experienced cardiologists, respectively. Although the total positive and negative numbers of the two diagnostic results were the same, their common positive or negative numbers are different. The fourfold table is shown in Table 1. The results from Kappa statistical analysis are as follows: the observation agreement *P*_0_ = 0.9583; the opportunity consistency *P*_*e*_ = 0.5703; Kappa coefficient *k* = 0.9030. Considering the sampling error, *u*-test was carried out for the assumption of *k* = 0. There is a strong consistency (*k* > 0.81) between the two methods due to the result of *u* = 13.47 larger than the standard normal quantile of 1.96. The inconsistent diagnostic results mainly occurred in the ST deviation near the threshold value of 0.1 mV. In this case, the repeatability of the 12-lead ECG detection is very poor, so it needs to be combined with clinical data detected by other equipment to make the diagnosis. In fact, many experienced cardiologists were also full of praise for the patterns displayed on the 3D heart model because of the obvious advantages in the determination of the region of myocardial ischemia. The results of a 69-year-old female patient are shown in Figure 3. She was diagnosed with extensive regional myocardial ischemia in the anterior, lateral, and inferior walls by an experienced cardiologist with her 12-lead ECG (see Figure 3(a)).  Figure 3(b) is the patient's pattern of myocardial ischemia displayed on a 3D heart model. Obviously, the region of myocardial ischemia is easy to be identified; even an experienced cardiologist cannot imagine such a vivid image in his brain.

**Figure 4:**
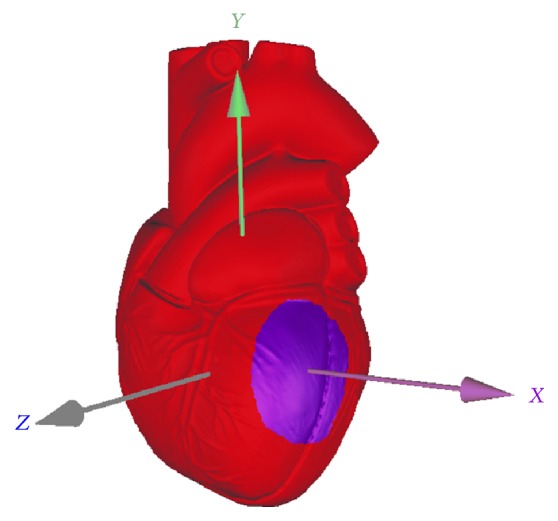
Myocardial ischemic region on the 3D heart model of a 60-year-old male patient.

Coronary angiography is the gold standard for the diagnosis of coronary artery stenosis. The left anterior descending, left circumflex, and right coronary artery supply blood to their respective regional myocardium. The corresponding myocardial region will be ischemic when a coronary artery stenosis occurs. It can be said that coronary angiography is one of the most reliable solutions to verify the display of myocardial ischemia on the 3D heart model. We conducted a total of 72 cases of comparative clinical trials, and the correct rate of display of myocardial ischemia on the 3D heart model was 98.4%. The myocardial ischemic region on the 3D heart model of a 60-year-old male patient is shown in Figure 4. Coronary angiography confirmed that the patient suffered from 40% stenosis of his left marginal artery and 50% of his left circumflex.

**Figure 5:**
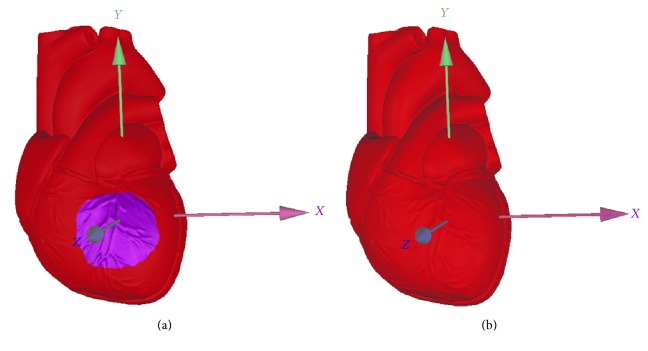
Comparison before and after the operation of coronary stent implantation. (a) Pattern before the stent implantation. (b) Pattern at two weeks after the stent implantation.

Coronary stent implantation is another scheme reliable to verify the display of myocardial ischemia on the 3D heart model. The myocardial tissue in ischemia due to coronary artery stenosis will restore the normal blood supply after stent implantation. It should be noted that the complete recovery of the normal blood supply to the cardiomyocytes is about two weeks after the stent is placed.  Figure 5(a) presented the region of myocardial ischemia on the 3D heart model of a 65-year-old male patient. Coronary angiography confirmed that his left anterior descending artery was stenosed by 70%. Two weeks after his stent implantation, the patient's pattern on the 3D heart model had been detected as shown in Figure 5(b). The myocardial ischemic region shown in Figure 5(a) completely disappeared here.

## 4. Conclusions

We have established a new method for the 3D visualization of the information of myocardial ischemia in the 12-lead ECG, which displays a realistic pattern of myocardial ischemia and does not require any restriction on the patient to be detected. Unlike earlier methods with many constraints, our method is based on the ST injury vector, which can accurately get the distribution range and the location of the myocardial ischemia in uniform and continuous distribution. The clinical validation protocol adopted was the comparison of the results obtained by our method with the diagnoses of experienced cardiologists and the results of coronary angiography. The experimental results indicate that our new method is successful, which can not only reduce the rate of misdiagnosis and missed diagnosis of medical professionals, but also make ordinary people diagnose their own myocardial ischemia like an experienced cardiologist. Our work and results have very good application prospect in medical clinical practice and health care field and are possible to make the 12-lead ECG into thousands of households.

There is a drawback to be further improved. At present, our method can only display a single continuous distribution of myocardial ischemia, but its actual distribution may be scattered in multiple regions.

## Figures and Tables

**Table 1:** Fourfold table of the diagnosis of myocardial ischemia.

		Experienced cardiologists		Total	Rate
		Positive	Negative		
Ordinary people	Positive	32	1	33	0.6875
	Negative	1	14	15	0.3125

	Total	33	15	48	
	Rate	0.6875	0.3125		

## References

[1] Boothroyd L. J., Segal E., Bogaty P. (2013). Information on myocardial ischemia and arrhythmias added by prehospital electrocardiograms. *Prehospital Emergency Care*.

[2] Frank A., Bonney M., Bonney S., Weitzel L., Koeppen M., Eckle T. (2012). Myocardial ischemia reperfusion injury: from basic science to clinical bedside. *Seminars in Cardiothoracic and Vascular Anesthesia*.

[3] Jennings R. B. (2013). Historical perspective on the pathology of myocardial ischemia/reperfusion injury. *Circulation Research*.

[4] Mozaffari Mahmood S., Yao L. J., Worku A., Babak B. (2013). Mechanisms of load dependency of myocardial ischemia reperfusion injury. *American Journal of Cardiovascular Disease*.

[5] Hutchison A. W., Malaiapan Y., Cameron J. D., Meredith I. T. (2013). Pre-hospital 12 lead ECG to triage ST elevation myocardial infarction and long term improvements in door to balloon times: the first 1000 patients from the MonAMI project. *Heart Lung and Circulation*.

[6] Van Deursen C. J. M., Blaauw Y., Witjens M. I. (2014). The value of the 12-lead ECG for evaluation and optimization of cardiac resynchronization therapy in daily clinical practice. *Journal of Electrocardiology*.

[7] Piers S. R. D., De Riva Silva M., Kapel G. F. L., Trines S. A., Schalij M. J., Zeppenfeld K. (2014). Endocardial or epicardial ventricular tachycardia in nonischemic cardiomyopathy? the role of 12-lead ECG criteria in clinical practice. *Heart Rhythm*.

[8] Perino A. C., Singh N., Aggarwal S., Froelicher V. (2014). The long-term prognostic value of the ST depression criteria for ischemia recommended in the universal definition of myocardial infarction in 43,661 veterans. *International Journal of Cardiology*.

[9] Perino A., Singh N., Aggarwal S., Perez M., Ashley E., Froelicher V. (2013). What is the prognostic value of the st depression criteria for ischemia recommended in the universal definition for myocardial infarction?. *Journal of the American College of Cardiology*.

[10] Andersen M. P., Terkelsen C. J., Struijk J. J. (2009). The ST Compass: spatial visualization of ST-segment deviations and estimation of the ST injury vector. *Journal of Electrocardiology*.

[11] Sangkachand P., Cluff M., Funk M. (2012). Detecting myocardial ischemia with continuous ST-segment monitoring: two case studies. *Heart & Lung*.

[12] Zizzo C., Hassani A., Turner D. (2008). Automatic detection and imaging of ischemic changes during electrocardiogram monitoring. *IEEE Transactions on Biomedical Engineering*.

[13] Greensite F., Huiskamp G. (1998). An improved method for estimating epicardial potentials from the body surface. *IEEE Transactions on Biomedical Engineering*.

[14] Trunk P., Mocnik J., Trobec R., Gersak B. (2007). 3D heart model for computer simulations in cardiac surgery. *Computers in Biology and Medicine*.

[15] Dankowski R., Baszko A., Sutherland M. (2014). 3D heart model printing for preparation of percutaneous structural interventions: description of the technology and case report. *Kardiologia Polska*.

[16] Liu K., Lu B., Zheng Z. (2015). GW26-e5394 3D heart model printing of complex congenital heart disease based on the low dose cardiac CT images: initial experience in China. *Journal of the American College of Cardiology*.

[17] Zhan Z.-Q., Wang C.-Q., Sclarovsky S., Nikus K. C., He C.-R., Shan M. (2013). ST-segment deviation pattern of takotsubo cardiomyopathy similar to acute pericarditis: diffuse ST-segment elevation. *Journal of Electrocardiology*.

[18] Resende L. O., Resende E. S., Andrade A. O. Assessment of the ST segment deviation area as a potential physiological marker of the acute myocardial infarction.

[19] Twerenbold R., Reichlin T., Abaecherli R. (2014). ST-segment deviation score in the early diagnosis of acute myocardial infarction. *ESC*.

[20] Cayla G., Silvain J., Collet J.-P., Montalescot G. (2015). Updates and current recommendations for the management of patients with non-ST-elevation acute coronary syndromes: what it means for clinical practice. *The American Journal of Cardiology*.

[21] Garg P., Nelson T., Sahu J., Sheridan P. (2015). Is this ST-elevation because of myocardial ischaemia or a Brugada pattern? An interesting case review. *Internal and Emergency Medicine*.

[22] Zhang Y.-J., Zheng W., Sun J., Li G.-L., Chi B.-R. (2015). Electrocardiogram score for the selection of reperfusion strategy in early latecomers with ST-segment elevation myocardial infarction. *Journal of Electrocardiology*.

[23] Man S., Rahmattulla C., Maan A. C. (2014). Acute coronary syndrome with a totally occluded culprit artery: relation of the ST injury vector with ST-elevation and non-ST elevation ECGs. *Journal of Electrocardiology*.

